# Trends in specialized low‐allergy infant formula dispensing in Ireland: 2016–2021

**DOI:** 10.1111/cea.14275

**Published:** 2023-01-25

**Authors:** Daniel O'Reilly, Robert Conway, Claire A. Murphy, Daniel Munblit, Patrick Fitzpatrick

**Affiliations:** ^1^ Department of Paediatrics Rotunda Hospital Dublin Ireland; ^2^ University College of Dublin School of Biomolecular and Biomedical Science UCD Dublin Ireland; ^3^ Health Service Executive Office of the National Clinical Director for Health Protection Dublin Ireland; ^4^ Department of Paediatrics RCSI Dublin Ireland; ^5^ Inflammation, Repair and Development Section, National Heart and Lung Institute, Faculty of Medicine Imperial College London London UK; ^6^ Department of Paediatrics and Paediatric Infectious Diseases Institute of Child's Health, Sechenov First Moscow State Medical University (Sechenov University) Moscow Russia; ^7^ Department of Paediatric Emergency Medicine Childrens Health Ireland Dublin Ireland

**Keywords:** cow's milk protein allergy, overdiagnosis, paediatrics, pharmacoepidemiology

**Key messages**

- We evaluated extensively hydrolysed, amino‐acid and soya formula prescriptions in databases covering ~10% Irish babies.
- Prescription of these low‐allergy formula products increased from 5.2% to 12.6% between 2016 and 2021.
- Formula feeding rates are higher in this population than in the total population of Ireland

To the Editor,

Cow's milk protein allergy (CMPA) is the overarching term for two distinct food allergy phenotypes, IgE‐mediated and non‐IgE‐mediated CMPA. While a diagnosis of IgE‐mediated CMPA can be confirmed on the basis of signs and symptoms with skin prick testing (SPT) or specific IgE testing, no such validated markers exist for non‐IgE‐mediated CMPA. Non‐IgE‐mediated CMPA is usually characterized by a wide range of behaviours (e.g., food refusal, irritability), gastrointestinal symptoms (e.g., change in bowel habit, bloody stools, vomiting, failure to thrive) and dermatological manifestations (e.g., non‐specific rashes and dermatitis).^1^ In formula‐fed infants, dietary exclusion of cow's milk protein is achieved with extensively hydrolysed formula (EHF) or amino acid‐based formula (AAF). In breastfed infants an exclusion diet is often recommended to the lactating mother, although evidence behind this approach is mixed.

The best available data for total IgE and non‐IgE‐mediated milk allergy prevalence is approximately 1%. The lack of objective diagnostic tools and dependence on broad, symptom‐based guidelines has led to concerns that non‐IgE CMPA is being over‐diagnosed.^2^ A recent secondary analysis of the Enquiring About Tolerance (EAT) trial demonstrated that in infants that were not exposed to cow's milk directly, almost 3 of 4 infants had symptoms of CMPA as defined by the international Milk Allergy in Primary care (iMAP) guidelines.^3^

Studies internationally have demonstrated an increase in number of prescriptions of EHF, AAF and soy‐based (SF) formula prescription rates over time.^4^ Ireland has very low rates of breastfeeding, which have been ascribed to a number of cultural and social phenomenon.^5^ While breastfeeding rates have improved, they remain lower than within comparable populations.^6^ This study examines time trends in the prescription of infant formulas marketed for CMPA in Ireland.

Data were extracted from the Primary Care Reimbursement Services (PCRS) database. This national database holds information on all publicly funded prescriptions under the General Medical Services scheme (GMS) and the Drugs Payment Scheme (DPS). Information request form for the data extraction was submitted to the PCRS. Data from 2016 to 2021 were requested, with inquiry targeting reimbursable infant formula items from the PCRS list of reimbursable items. Data were provided on the number of unique infants aged 6 months or less who received a prescription for either an EHF, AAF or SF in this timeframe.

GMS is a reimbursement scheme in Ireland whereby registered lower income families can avail of general practitioner and pharmaceutical services free (or very low cost) at point of use. While the income threshold for eligibility varies (depending on age at time of application, and whether the applicant is a single person or has a family), the weekly income of those eligible for the scheme ranges from €38 to €298.

DPS is a non‐means tested, opt‐in scheme, where an individual or family has to pay no more than a monthly threshold amount in a calendar month for approved drugs (between €114 and €144 during the study period). Existing regulations do not allow for an individual to be a part of both schemes simultaneously.

There were 21,867 infants registered for the GMS during the study period with a further 5967 infants registered for DPS. All infant formulas which were both (1) reimbursable and (2) marketed primarily for CMPA were included. Live birth rates were derived from perinatal and Irish maternity statistics from 2016 to 2021. Breastfeeding rates on discharge from maternity hospitals were extracted from the annual perinatal statistics report (2016–2018) and the Irish Maternity Indicator System (IMIS) national report (2019–2020).

A Joinpoint regression analysis was used to calculate the annual percent change (APC) in formula prescribing. This has been utilized previously in drug utilization studies to demonstrate changes in prescribing trends over time.^7^ Rates of unique infants prescribed a formula product per 1000 infants registered for a scheme were used. A permutation test model was used, with significance level set at .05 to calculate 95% confidence intervals (CI). No minimum joinpoint number was set as only six data points were utilized.

Formal research ethics approval was not sought for this study since human participants were not directly involved in this secondary data analysis. All the data collected for this study were publicly available. Data collected are presented in Table 1.

**TABLE 1:** Number of unique infants eligible/registered for the schemes, total live births and breastfeeding rates in 2016–2021

Year	2016	2017	2018	2019	2020	2021	Totals
*GMS*							
Total eligibility	4618	4329	3744	3746	2819	2611	21,867
AAF	101	109	114	115	109	106	654
EHF	47	54	76	185	151	105	618
Soy	24	20	12	9	9	9	83
Any CMP‐free formula	172	183	202	309	269	220	1355
Any CMP‐free formula, %	3.7	4.2	5.4	8.2	9.5	8.4	6.2
*DPS*							
Total eligibility	1171	1041	853	893	762	977	5697
AAF	113	120	128	115	127	134	737
EHF	15	8	56	85	122	98	384
Soy	0	0	1	0	0	0	1
Any CMP‐free formula	128	128	185	200	249	232	1122
Any CMP‐free formula, %	10.9	12.3	21.7	22.4	32.7	23.7	19.7
*Combined GMS and DPS*							
Total eligibility	5789	5370	4627	4639	3581	3588	27,564
Any CMP‐free formula	300	311	387	509	518	452	2477
Any CMP‐free formula, %	5.2	5.8	8.4	11	14.5	12.6	9
*Breastfeeding and live births*							
Breastfeeding rate, %	59.9	59.8	60.4	63.3	58.5	NR	
Live births	63,870	61,854	61,061	59,352	56,835	58,443	361,415

*Note*: Breastfeeding is defined as ‘any breastfeeding on discharge from maternity unit’. Breastfeeding data from 2021 is yet to be compiled.

Abbreviations: AAF, amino acid‐based formula; DPS, Drugs Payment Scheme; EHF, extensively hydrolysed formula; GMS, General Medical Services scheme; NR, not reported.

For the two schemes over 6 years, a total of 8.96% of registered infants were prescribed a specialized formula. The percentage of infants that have received a prescription for a formula product for the treatment of CMPA significantly increased in both schemes from 2016 to 2021 (Figure 1A,B). The APC on the GMS scheme for any CMPA formula product was 21.5% (95% CI: 8.9%, 35.5%). The APC for those on the DPS scheme was 21.6% (95% CI: 4.3%, 41.7%).

**FIGURE 1:**
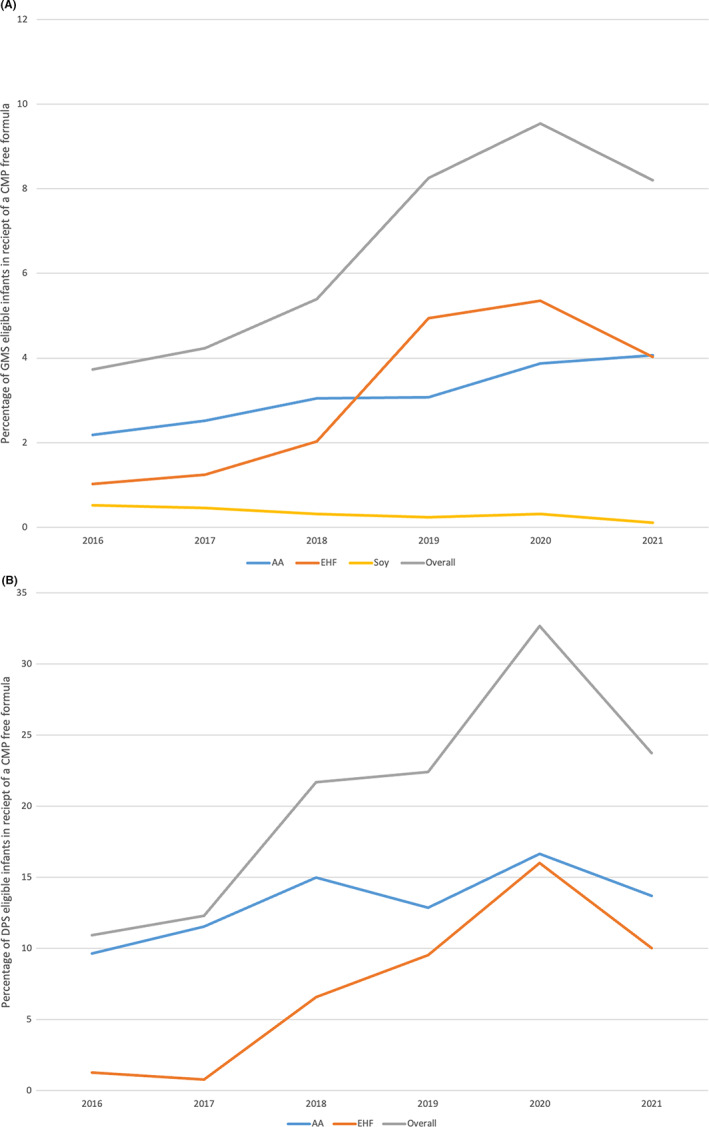
(A) Percentage of unique infants prescribed specialized formula in the General Medical Services (GMS) scheme dataset. (B) Percentage of unique infants prescribed cow's milk free formula in the Drugs Payment Scheme (DPS) dataset. Soy not included in DPS graphic as only 1 individual prescribed over period examined.

The absolute percentage of infants on both schemes who were prescribed a formula product for the treatment of CMPA increased from 5.2% of infants in 2016 to 12.6% in 2021. For infants who were registered for GMS alone the rate increased from 3.7% to 8.4%.

The predominant type of formula dispensed for the treatment of CMPA over the study period was AAF as opposed to EHF. Prescription rates for both EHF and AAF increased during the study period. However, from 2018 to 2019 EHF prescription rates increased much more rapidly. Infants on the GMS scheme had a 13.4% (95% CI: 9.6%, 17.3%) APC in their utilization of AAF as compared with a 41.4% (95% CI: 11.8%, 78.8%) APC in utilization of EHF.

There was no statistically significant increase in infants who received prescriptions through the DPS for AAF with an APC of 8.1% (95% CI −1.8%, 18.9%). However, a substantial APC increase was noted in EHF of 75.9% (95% CI 9.1%, 183.6%) which was responsible for the demonstrated increase in overall formula prescriptions (Figure 1B).

Utilization of formula for the treatment of CMPA in the population studied was high within the time frame of the past 5 years. For the entirety of the study period, 9% of registered infants were in receipt of a specialized formula while the estimated prevalence of CMPA in Europe is approximately 1%.^8^

AAF was the most widely utilized type of specialized formula in the past 5 years. Guidelines recommend that EHF should be trialled for 4–6 weeks initially, and if symptoms do not resolve, then an AAF should be utilized instead.^1^ Given the lack of evidence for superiority of AAF versus EHF, non‐resolution of symptoms may instead represent a misdiagnosis of CMPA as opposed to a failure to respond to initial treatment, with consequent escalation of therapy.

Soy‐based formula prescription rates reduced throughout the study period. This is potentially related to their prevailing pattern of use in Ireland. While soy‐based formulas are marketed for the treatment of CMPA, in Ireland their primary use is for infants with classical galactosaemia identified on newborn population screening or on selective screening of the endogamous Irish Traveller population.^9^ Concerns regarding phytoestrogen content of soy‐based formula and parental preferences may also play a role.

This study has strengths and limitations. The analysis of national specialized formula prescribing trends is unique in examining prescriptions for individual infants under 6 months old in a country with low breastfeeding rates. The prescribing data for over 27,000 unique infants made it possible to demonstrate a statistically significant rising trend in prescriptions over a multi‐year period. The study population included between 10% and 11% of the national population. Specialized formula prescribing trends were similar for both the more economically deprived infant group (GMS) and the rest of the population infant group (DPS).

Those populations that were more likely to formula feed were more likely to be registered for either GMS or DPS compared to the general population. The GMS scheme is means‐tested and represents a socioeconomically deprived cohort which in other studies has a higher tendency to formula feed.^6^ While DPS is non‐means tested, families have to register for the scheme and are more likely to do this if there are existing healthcare costs in the household (e.g., specialized formula prescriptions). Anecdotally, these formulas may be prescribed at a higher rate to low‐income families as a means to provide formula at a lower cost.

In conclusion, specialized formula prescription rates for individual infants in Ireland in this large national dataset has increased since 2016. This trend has been driven primarily by the increased utilization of EHF formulas, and continuing steady increases in AAF prescriptions. This trend has occurred out of proportion to the prevalence of the condition, and further research will be required to elucidate its causes.

Potential hypotheses to explain the observed trend include an increasing adherence to European guidelines regarding both CMPA and reflux driving an increase in EHF prescriptions at an earlier stage in the treatment pathway; and ‘therapeutic creep’ where babies with symptoms consistent with normal infancy are increasingly being treated as ‘mild/moderate CMPA’.^1^ Other hypotheses include increased recognition of other allergic conditions such as Food Protein‐Induced Enterocolitis Syndrome or Eosinophilic Oesophagitis; however, given their relative rarity in this age group, it is difficult to imagine that they are entirely responsible for the observed increase over time.

## AUTHOR CONTRIBUTIONS

DOR, CAM, RC drafted the initial manuscript. DOR and RC completed statistical analysis and obtained relevant data from PCRS. DOR, RC and PF developed the initial hypothesis and study design. DOR, RC, CAM, DM and PF edited manuscript and reviewed prior to submission.

## CONFLICT OF INTEREST

There is no conflict of interest to declare.

## Data Availability

The data that support the findings of this study are available by request from the corresponding author or through request via the Primary Care Reimbursement service (https://www.hse.ie/eng/staff/pcrs/online‐services/)
